# Structure-Function Studies of DNA Binding Domain of Response Regulator KdpE Reveals Equal Affinity Interactions at DNA Half-Sites

**DOI:** 10.1371/journal.pone.0030102

**Published:** 2012-01-23

**Authors:** Anoop Narayanan, Lake N. Paul, Sakshi Tomar, Dipak N. Patil, Pravindra Kumar, Dinesh A. Yernool

**Affiliations:** 1 Department of Biological Sciences, Purdue University, West Lafayette, Indiana, United States of America; 2 Bindley Bioscience Center, Purdue University, West Lafayette, Indiana, United States of America; 3 Department of Biotechnology, Indian Institute of Technology, Roorkee, India; Institute of Enzymology of the Hungarian Academy of Science, Hungary

## Abstract

Expression of KdpFABC, a K^+^ pump that restores osmotic balance, is controlled by binding of the response regulator KdpE to a specific DNA sequence (*kdpFABC_BS_*) via the winged helix-turn-helix type DNA binding domain (KdpE_DBD_). Exploration of *E. coli* KdpE_DBD_ and *kdpFABC_BS_* interaction resulted in the identification of two conserved, AT-rich 6 bp direct repeats that form half-sites. Despite binding to these half-sites, KdpE_DBD_ was incapable of promoting gene expression *in vivo*. Structure-function studies guided by our 2.5 Å X-ray structure of KdpE_DBD_ revealed the importance of residues R193 and R200 in the α-8 DNA recognition helix and T215 in the wing region for DNA binding. Mutation of these residues renders KdpE incapable of inducing expression of the *kdpFABC* operon. Detailed biophysical analysis of interactions using analytical ultracentrifugation revealed a 2∶1 stoichiometry of protein to DNA with dissociation constants of 200±100 and 350±100 nM at half-sites. Inactivation of one half-site does not influence binding at the other, indicating that KdpE_DBD_ binds independently to the half-sites with approximately equal affinity and no discernable cooperativity. To our knowledge, these data are the first to describe in quantitative terms the binding at half-sites under equilibrium conditions for a member of the ubiquitous OmpR/PhoB family of proteins.

## Introduction

Bacteria make extensive use of two-component signal transduction systems (TCS) to respond to changes in the external environment and to internal cues [1], [2], [3]. Generally, TCS consist of a multi-domain membrane-bound sensor histidine kinase and a response regulator (RR) that launches a cellular response upon stimulation. Histidine kinases perceive chemical or physical stimuli from three potential directions with respect to the membrane: from outside, inside, or within the membrane itself [4]. These stimuli are converted to signals via a series of phosphotransfer events involving autophosphorylation, transphosphorylation, and dephosphorylation reactions: The first two steps constitute the activation process of signaling, and the latter involves termination by dephosphorylation [3].

RRs catalyze the transfer of a high-energy phosphoryl group from a histidine on activated kinases to a conserved aspartate residue in the receiver domain of the RR [5], [6]. In addition to having a universal phosphor-accepting receiver domain (RD), many RRs contain a variety of output domains that either bind DNA, RNA, protein, and other ligands or possess enzymatic activity [7]. Although a variety of functions have been described for these output domains, many (63%) bind DNA to regulate transcription [8]. Two architectural motifs prevail in DNA binding domains (DBDs): the winged helix-turn-helix (wHTH), which is exemplified by the OmpR/PhoB family of proteins [9] that constitute >60% of all DNA binding RRs, and the classic helix-turn-helix motif observed in the NarL/FixJ [10] and NtrC/DctD families [11].

One of the major responses to hyperosmotic stress in bacterial cells is the accumulation of K^+^ to restore hydration of cytoplasmic contents [12], [13]. When cells are under stress and the constitutively active Kup and Trk transporter systems are unable to meet the demand for K^+^, cells can produce the high-affinity KdpFABC transporter to reinstate homeostasis [14], [15]. Synthesis of hetero-oligomeric KdpFABC, a P-type ATPase, is controlled by a widely distributed TCS consisting of the transmembrane histidine kinase KdpD and its cognate RR, KdpE [16], [17]. Topologically complex and distinct from other histidine kinases, KdpD has a large N-terminal input domain attached to four transmembrane segments followed by a C-terminally located histidine kinase domain [18]. The minimal exposure of residues to the extracellular milieu and the presence of large N- and C-terminal domains facing the cytoplasm support the hypothesis that KdpD perceives signals from either the membrane or the cytoplasm. Although the precise signal(s) remain unknown, KdpD integrates multiple inputs, including drops in extracellular K^+^ concentration [19], changes resulting from ionic hyperosmolarity [20], changes in membrane lipid composition [21] and ATP levels [22], to activate KdpE by phosphorylation [23]. Phosphorylated KdpE (KdpE∼P) interacts as a cis-acting element in the promoter region, resulting in transcription of the *kdpFABC* operon [24]. The interactions of the DBD of KdpE (KdpE_DBD_) with DNA are most likely mediated by a wHTH motif.

Structures of five full-length OmpR/PhoB family proteins have been determined in addition to fourteen of DBDs, and a large number of RDs in inactive and active conformations. The RDs often form dimers in crystal structures involving the α4-β5-α5 interface [5], [6], an interface thought to represent the activated conformation [25]. The structural and biochemical data suggest two subgroups within the family based on the oligomerization state of the RRs after phosphorylation: (i) those that form dimers before binding DNA as exemplified by PhoB [26] and (ii) and other OmpR-like proteins that form stable dimers only after binding DNA. Irrespective of their oligomerization state members of this family recognize direct (imperfect) repeat sequences that form a pair of half-sites that constitute a single recognition site. Both single and multiple recognition sites that are either adjacent or well-separated from each other have been documented. Hierarchical binding as well as cooperativity between recognition sites also have been described [27]. However, two basic questions remain largely unaddressed as suggested by Kenney and others [28]: What are the protein binding affinities to each half-site within a single recognition site? Is there any cooperativity between these half-sites? The questions have to be tackled at two levels because the known interactions between RDs are expected to complicate the analysis. First, interactions between the isolated DNA binding domains with DNA have to be characterized which will define the contributions to binding independent of the receiver domains, followed by quantitative analysis of the interactions using full-length proteins both in inactive and active states. Focusing on the DNA binding domain, this study describes the comprehensive characterization of interactions between KdpE_DBD_ and its recognition site (*kdpFABC_BS_*) by: identifying the two 6 bp direct DNA repeats; determining the X-ray structure of KdpE_DBD_ and identifying residues involved in DNA binding by mutagenesis; and quantitative analysis of DNA protein interactions by analytical ultracentrifugation establishing equal affinity binding to half-sites with no detectable cooperativity within the limits of the data.

## Methods

### Protein expression and purification

DNA sequences encoding *E. coli* KdpE (*Escherichia coli* str. K-12 substr. MG1655; accession no: AAC73788; residues 1–225) and KdpE_DBD_ (residues 124–225) were amplified by PCR (for primers used see Table S1) and cloned into pHisP1 vector. KdpE_DBD_ was expressed as a fusion protein containing an N-terminal His-tag and tobacco etch virus (TEV) protease site. Protein production in *E. coli* BL21 (DE3) grown at 37°C in Luria-Bertani medium was induced by adding 0.5 mM isopropyl β-D-1-thiogalactopyranoside to cultures at OD_600_ of 0.6. After overnight incubation at 18°C, the cells collected by centrifugation from 1 L of culture were suspended in 45 ml PBSK buffer (50 mM sodium phosphate pH 7.5, 140 mM NaCl, 10 mM KCl, 10% v/v glycerol) containing 0.1 mg DNAse and protease inhibitor cocktail (Sigma Aldrich Co.). Cells were lysed by three passages through a cell disruptor (Avestin Corp.), and the His-tagged proteins were isolated from clarified lysate by binding to a 5 ml Ni-NTA Hi-Trap column (GE Healthcare). The column was washed with 20 and 10 column volumes of PBSK buffer containing 10 and 20 mM imidazole, respectively, and bound protein was eluted in PBSK buffer containing 200 mM imidazole. All steps were performed at a flow rate of 2.0 ml/min at room temperature (25°C). After elution, the protein was treated overnight at room temperature with a 1∶100 mass ratio of TEV protease to remove the His-tag. The final step of purification was achieved by size-exclusion chromatography on a HiLoad 16/60 Superdex 200 column (GE Healthcare) in 10 mM HEPES pH 7.5, 150 mM KCl at a flow rate of 1.0 ml/min. Analytical size-exclusion chromatography was performed using a 10/300 GL Superdex 200 column (GE Healthcare) in the same buffer. The protein concentration was quantified by measuring absorbance at 280 nm and using extinction coefficients of 18,450 and 9,960 M^−1^ cm^−1^ for purified KdpE and KdpE_DBD_, respectively.

### β-Galactosidase assay

For the *in vivo* signaling studies, the following were constructed using primers listed in Table S1: *kdpD* controlled by a tetracycline-inducible promoter in pTEVGH11 vector (*amp* ^r^), and *kdpE_DBD_* and *kdpE* genes (including point mutants of the latter) fused to a phage T7 promoter in pRSFD1 vector (Novagen Inc.; *kan^r^*). Pairs of plasmids encoding KdpD kinase and KdpE_DBD_, KdpE, or their mutants were co-transformed into *E. coli* RH003 strain [(Δ*kdpDE*, *kdpFABC* promoter-*lacZ^+^* fusion, *kdp ABCDE81*, Δ *(lac-pro) ara, thi*); a gift from Drs. Altendorf and Jung [29]] and then were selected on KLM medium (1% KCl, 1% casein hydrolysate, 0.5% yeast extract) supplemented with ampicillin and kanamycin. Cultures were grown to mid-logarithmic phase in K0 or K10 media as per the protocol described by Heermann and others, [29] and *kdpFABC* expression was monitored by measuring the β-galactosidase activity expressed as Miller units.

### Electrophoretic Mobility Shift Assay (EMSA)

A 30 bp double-stranded DNA representing the binding site (*kdpFABC_BS_*) for KdpE [29] synthesized and purified by HPLC by IDT Inc. (www.idtdna.com) for use in EMSA and AUC analyses. This DNA contains the 23 bp CATTTTTATACTTTTTTTACACCCCGCCCG sequence that was protected from DNAse-I digestion in footprinting analysis [24]. Table S2 provides the list of oligonucleotides used for EMSA analysis. Pairs of complementary primers were annealed to produce double-stranded DNA molecules and 2 µl of 5 pmoles/µl of DNA was used in a 10 µl reaction which was loaded on gels for EMSA analysis. Mixtures of protein and double-stranded DNA at indicated molar ratios in EMSA buffer (10 mM Tris pH 8.0, 5% glycerol, 50 mM NaCl, 0.1% Triton ×100, 10 mM dithiothreitol, and 1 mM EDTA) were separated on a 6% acrylamide gel using TBE buffer (89 mM Tris base, 89 mM boric acid, 2 mM EDTA). The DNA in the gel stained with ethidium bromide was imaged using the Kodak Image Station 2000R.

### Crystallization, data collection, structure determination, and refinement

Purified KdpE_DBD_ concentrated to 18 mg/ml and used in sitting drop format yielded crystals when mixed in a 1∶1 ratio with well solution containing 1.5 M lithium sulfate and 0.1 M HEPES pH 7.5. Addition of dioxane (5%) yielded larger crystals. For cryoprotection, crystals briefly exposed to well solution containing 20% glycerol were mounted in cryoloops prior to collection of X-ray diffraction data. The data collection statistics are shown in Table 1. The diffraction data were processed using the *HKL*-2000 package (http://www.hkl-xray.com/). Initial phases were obtained by the molecular replacement method [30] using MOLREP with the structure of the DBD of RegX [PDB ID: 2OQR] as the search model. Model building was conducted in manual mode in Coot [31], followed by automated refinement in Refmac 5.2 [32]. The final model for KdpE_DBD_ contains residues 125 and 225 of full-length protein. The quality of the model was evaluated using ProCheck.

**Table 1:** Crystallographic data and results of refinement.

Crystallographic data	
Space group	*P*4_3_2_1_2
Wavelength	1.5418
Resolution	50–2.5
Cell dimensions	
*a* (Å)	36.4
*b* (Å)	36.4
*c* (Å)	138.4
α (°)	90.00
*β* (°)	90.00
γ (°)	90.00
Unique reflections	3378 (389)
Completeness (%)	95.0 (77.7)
R_sym_(%)^a^ (Last Shell)	6.5 (10.2)
I/σ (Last shell)	19.0 (9.1)
Multiplicity (Last shell)	4.1 (3.6)
Refinement	
Water molecules	23
Resolution range (Å)	35.0–2.5
*R-*work (%)	23.6
*R-*free (%)	28.4
Average *B*-factors (Å^2^)	17.5
rmsd on bond lengths (Å)	0.01
rmsd on bond angles (?)	1.66
Ramachandran plot (%)	
Preferred	84.0
Allowed	16.0
Outliers	0.0

a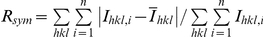.

b *R*_work_ = ∑‖*F*_o_|−|*F*_c_‖/∑|*F*_o_| for reflections contained in the working set, and *R-*free = ∑‖*F*_o_|−|*F*_c_‖/∑|*F*_o_| for reflections contained in the test set held aside during refinement. |*F*_o_| and |*F*_c_| are the observed and calculated structure factor amplitudes, respectively.

### Analytical ultracentrifugation

*kdpFABC_BS_* DNA, mutant versions with dinucleotide substitutions (Table S2, *kdpFABC_BS_—1* and *kdpFABC_BS_—7* which are incapable of binding KdpE_DBD_ at half-sites S-1 and S-2 respectively), and purified KdpE_DBD_ were used for this analysis. Sedimentation velocity (SV) experiments were conducted at 50,000 rpm on Beckman-Coulter analytical ultracentrifuges, XLA and XLI (Beckman-Coulter, CA) using absorbance optics at 280 and 260 nm. DNA-protein complexes, DNA and protein alone were characterized at 20°C in 50 mM HEPES, pH 7.4 buffer containing 150 mM KCl and 1 mM EDTA by titrating double-stranded DNA at 0.5 µM with varying concentrations of KdpE_DBD_ (0 to 16 µM). Individual components of the complex were analyzed as follows: The solvent density (1.00166 g ml^−1^), viscosity (0.01016 poise), and partial specific volume (0.7438 ml g^−1^) of KdpE_DBD_ were calculated using SEDNTERP v. 1.09 (http://www.rasmb.bbri.org/rasmb/windows/sednterp-philo). The sedimentation coefficients (not corrected for 20°C and water) and apparent molecular weights were calculated from size distribution analyses, c(s), using SEDFIT v. 12.43 [33].

Sedimentation equilibrium (SE) experiments were conducted at 20°C using a 2-channel centerpiece placed in an AN-60 Ti rotor spun at speeds of 9,000, 19,800, and 34,000 rpm. The molar ratios of protein to DNA used to determine the *K_d_* and molecular weight of the complex were 0.5∶1, 1∶1, 1∶2, 1∶5, and 1∶10. Absorbance scans at 260 and 280 nm were taken at 2 hour intervals for a total of 60 hours. The samples were tested for equilibrium conditions using SEDFIT v 12.43. The calculations of the molecular weight of the complex and equilibrium constants were conducted using SEDPHAT v 8.62 [34]. A major factor influencing the determination of stoichiometry of the KdpE_DBD_-*kdpFABC_BS_* association is the contribution of partial specific volume (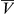, vbar) of the DNA-protein complex to its molecular weight. The 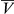 of the DNA was calculated from the GC content of the DNA (Table S3) [35]. The 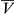 of the protein was calculated from its primary sequence using SEDNTERP. The 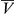 of the protein:DNA complex was estimated using the following equation: 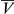
_complex_ = (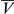
_DNA_+(R *
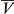
_protein_))/(1+R), where R is the ratio of protein to DNA masses in the complex [36]. The KdpE_DBD_ extinction coefficients used in these experiments were ε_280_ 9,960 M^−1^ cm^−1^ and ε_260_ 6,000 M^−1^ cm^−1^ determined using SEDNTERP. Sedimentation equilibrium of KdpE_DBD_ and *kdpFABC_BS_* DNA alone were also performed concurrently with the complexes. For the determination of the *K_d_*s and complex stoichiometry, the extinction coefficient of the DNA at 260 nm was estimated using IDT website (http://biophysics.idtdna.com), while at 280 nm, the extinction coefficient was calculated using SEDPHAT using the monomer-dimer self-association model in which the log(Ka) was set to 0; which in effect makes it a single species analysis with the benefits of mass conservation and fitting the loading concentrations. The extinction coefficient values determined were: ε_260_ 469,009 M^−1^ cm^−1^ and ε_280_ 259,485 M^−1^ cm^−1^. The experimentally determined DNA partial specific volume (0.57 ml/g) using sedimentation equilibrium (Single Species with Mass Conservation) agreed with value obtained from the GC method (0.59 ml/g) outlined by Kar, *et. al*, 2001. These values of partial specific volumes when used to determine masses of the DNA and DNA-protein complexes resulted in differences less than the error limits of calculations. For the *K_d_* and complex stoichiometry calculations these values were not allowed to float in SEDPHAT. The single non-interacting species model in SEDPHAT was used to calculate the molecular weights of the complexes [36].

To assess the interaction of KdpE_DBD_ at the half-sites S1 and S2, double-base substitutions at the individual sites were made, resulting in *kdpFABC_BS_—1* and *kdpFABC_BS_—7*, in which only S2 and S1, respectively, are competent to bind. KdpE_DBD_ binding to these sites was analyzed using SE experiments at 13,800, 26,500, and 45,000 rpm using a 6 channel centerpiece. The molar ratios were 1∶1, 1∶4, and 1∶16 DNA to protein. Data were collected at 2 hour intervals at 260 and 280 nm for 65 hours. The extinction coefficients for the DNA mutants were ε_260_ 474,268 M^−1^ cm^−1^ and ε_280_ 261,595 M^−1^ cm^−1^ for *kdpFABC_BS_—1* and ε_260_ 472,138 M^−1^ cm^−1^ and ε_280_ 260,661 M^−1^ cm^−1^ for *kdpFABC_BS_—7*. The SE data were sorted using SEDFIT, and SEDPHAT was used to globally fit the 260 and 280 nm data. The heterogeneous ABB (with symmetrical sites and macroscopic K) model was used to analyze the *K_d_* of the *kdpFABC_BS_* DNA and KdpE_DBD_ samples, and the heterogeneous AB model with mass conservation was used for the double-base mutants [37], [38]. The ABB model (with symmetrical sites and macroscopic K) in SEDPHAT gives a macroscopic *K_d_* for the first binding event, followed by a second *K_d_* for the preformed 1∶1 complex associating with its second binding partner. Error bars for the calculated *K_d_s* were generated using F-statistics with 1σ confidence interval.

## Results and Discussion

### Characterization of KdpE_DBD_

**Figure 1:**
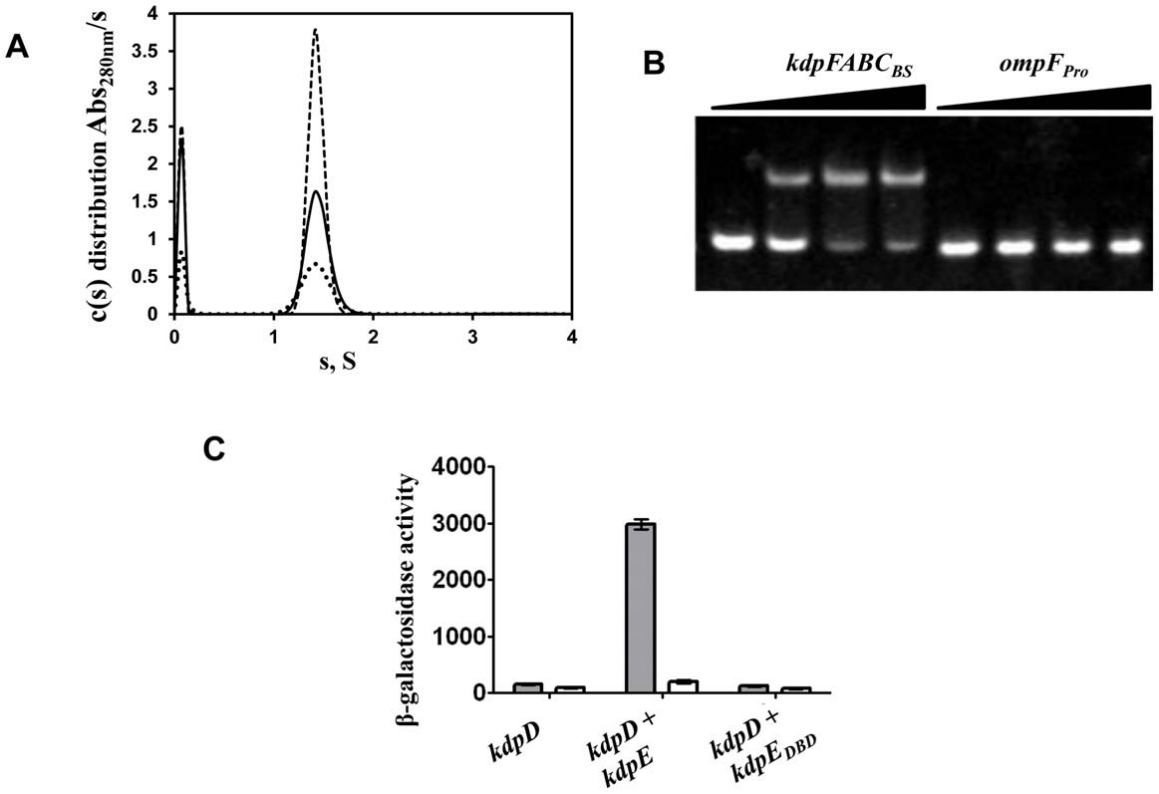
Biochemical and functional characterization of KdpE_DBD_. **A.** Sedimentation velocity analysis of the KdpE_DBD_ to detect self-association. The c(s) distribution of the KdpE_DBD_ at 21 (dots), 42 (solid line), and 84 µM (dashes) shows a single species of 1.4 S. No concentration-dependent formation of higher-order species was observed. **B.** Interaction of KdpE_DBD_ protein with *kdpFABC_BS_* and *ompF_Pro_* DNA sequences analyzed by EMSA. The triangles represent increasing molar ratios of 1∶0, 1∶1, 1∶2, and 1∶3 of DNA to purified KdpE_DBD_. The lower and upper bands represent free DNA and DNA-KdpE_DBD_ complex, respectively. **C.** *In vivo* analysis of expression of the β-galactosidase gene fused to *kdpFABC_Pro_*. *E. coli* RH003 cells lacking the histidine kinase (*kdpD*) and RR (*kdpE*) were used to express full-length KdpD alone as well as KdpD combined with KdpE or KdpE_DBD_. As described in the methods, the cells were grown in K0 (▪) and K10 (□) media prior to analysis of gene expression. Growth in K0 medium mimics stresses resulting from external K^+^ depletion. The β-galactosidase activity expressed as Miller units represents the mean of three independent experiments; error bars represent standard error.

Purified KdpE_DBD_ after removal of octa-histidine tag showed a single band in SDS-PAGE analysis and a unique peak in size exclusion chromatography (Fig. S1). SV analysis (Fig. 1A) revealed a single species with a sedimentation coefficient of 1.4 S, even at protein concentrations as high as 84 µM: The best-fit frictional ratio obtained from the analysis returns an estimated molecular mass of 12.1 kDa for this species, suggestive of a monomer, an interpretation that is supported by the position of elution in size exclusion chromatography when compared to elution positions of protein standards with known molecular mass (Fig. S1). The monomeric state of KdpE_DBD_ is consistent with previous studies on DBDs of other OmpR/PhoB family members. Analysis of the interaction of KdpE_DBD_ with its cognate recognition site *kdpFABC*_BS_ showed a significant mobility shift of DNA (Fig. 1B). In contrast, no changes in mobility of *ompF_Pro_* DNA were observed. *ompF_Pro_* represents the recognition site of OmpR, a RR belonging to the same protein family as KdpE (Fig. 1B). This lack of interaction with *ompF_Pro_* demonstrates that KdpE_DBD_ has inherent specificity towards *kdpFABC*_BS_.

In RH003 cells, which were engineered by removing the *kdpD* and *kdpE* genes and by fusing a β-galactosidase reporter gene to the *kdpFABC* promoter, β-galactosidase production was observed under K^+^ limiting conditions only when wild-type KdpD and KdpE were co-expressed (Fig. 1C). However, co-expression of KdpD and KdpE_DBD_ failed to elicit β-galactosidase production, indicating the need for the RD of KdpE for gene transcription despite KdpE_DBD_ retaining its primary biochemical function of specific binding to *kdpFABC*_BS_. The inability of KdpE_DBD_ to promote gene expression is similar to that of the DBD of OmpR [39] and is distinct from the DBD of PhoB, which expresses the reporter gene by binding with 7-fold greater affinity than unphosphorylated PhoB to the *pho* box sequence [40]. In full-length PhoB and MtrA [41], the interactions of the RD with DBD sterically prevent the latter from binding to DNA. This inhibition is relieved by phosphorylation of the RD [40]. A different route to gene regulation was proposed for OmpR wherein DBD of OmpR binding to DNA at low affinity was incapable of transcriptional initiation. The full-length protein binds as a monomer to DNA which stimulates phosphorylation and subsequent dimerization via RDs only in the DNA-bound state resulting in high-affinity interactions [27], [28]. The data suggests KdpE_DBD_ may have similarities to OmpR-type of interactions.

### X-ray structure of the KdpE_DBD_ protein

**Figure 2:**
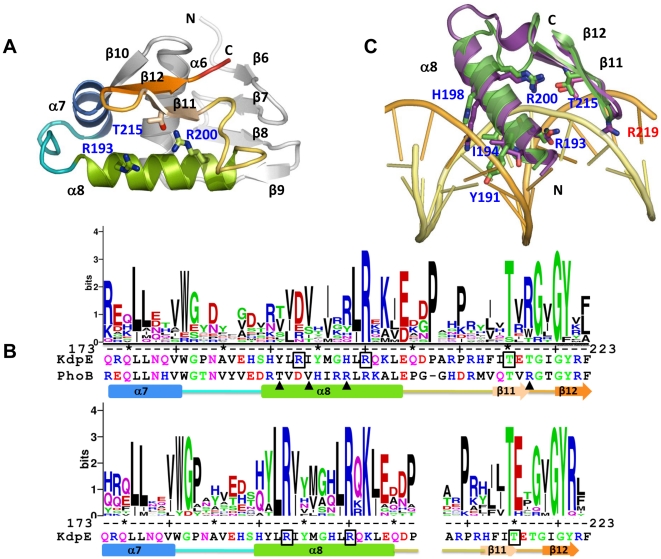
Structure of KdpE_DBD_. **A.** A cartoon representation of a molecule showing the wHTH motif in progressive coloring; the rest is in gray. To maintain continuity with the structure of the N-terminal receiver domain of KdpE [25], the β-strands and α-helices of KdpE_DBD_ are labeled starting with β-6 and α-6. The side chains shown in stick representation are residues R193 and R200 in α8 and T215 in β11 targeted for mutagenesis. N and C refer to the amino- and carboxyl- termini. **B.** Conservation of the sequence in the wHTH motif across members of the OmpR/PhoB family (upper panel) and between KdpE orthologs (lower panel) presented in logo format derived from multiple sequence alignments [61]. The Y-axis represents sequence conservation in bits. The residues targeted for mutagenesis in KdpE are boxed, the triangles represent residues involved in base specific interactions in PhoB-DNA complex (PDB code: 1GXP), and the residue numbering is that of KdpE sequence. Shown below the logo representation are the sequences of the wHTH motif of KdpE and PhoB (upper panel) and that of KdpE in the lower panel. The gap in the lower panel represents a three residue insertion in few of the KdpE orthologs used in sequence alignment. The schematic of the secondary structure was derived from the structure of KdpE_DBD_. **C.** Superposition of KdpE_DBD_ onto the structure of PhoB bound to DNA (PDB code: 1GXP). Only wHTH motifs of KdpE_DBD_ and chain A of PhoB in 1GXP and part of the DNA are shown. The coloring scheme: green, KdpE_DBD_; purple, PhoB and yellow/orange, DNA strands. The following side chains of residues of PhoB (and in parenthesis equivalent residues in KdpE_DBD_ labeled in blue) are shown as sticks: T194 (Y191), V197 (I194), R201 (H198) and R219 (T217, not shown), R203 (R200) and T217 (T215) and D196 (R193). Residues T194, V197, R201 and R219 (that penetrates the minor groove is labeled in red) of PhoB have been shown to be form base specific interactions.

To determine the structure of KdpE_DBD_, X-ray diffraction data was collected up to 2.5 Å resolution. Assuming one molecule of KdpE_DBD_ with mass of 12022 Da per asymmetric unit in the *P*4_3_2_1_2 space group, the calculated Matthews coefficient (*V*_M_) value and solvent content corresponds to 2.10 Å^3^ Da^−1^ and 41% respectively. The KdpE_DBD_ structure was solved by molecular replacement method and refined to an R-work of 23.6% and R-free of 28.4% (Table 1). Overall, the structure of KdpD_DBD_ resembles that of other members of the OmpR/PhoB family of proteins: It has a central three-helix core (α6-α7-α8) bookended by two β-sheets containing four and two strands at the N- and C-termini, respectively (Fig. 2A). The C-terminal pair of anti-parallel β-strands (β11–β12) that form a β-hairpin structure constitutes the wing of the wHTH motif. Figure 2B shows the sequence conservation in the wHTH motif in logo representation. The logos were created from a multiple sequence alignment made using sequences from known 3D structures of DBDs from OmpR/PhoB family members (Fig. S2) and from orthologs of KdpE respectively. The 15-residue-long, solvent exposed helix α8 of KdpE contains five positively charged residues that can potentially interact with DNA. The variations in sequences between α8 helices of PhoB and KdpE possibly reflect the differences in recognition sites of the two proteins.

Comparison of KdpE_DBD_ to DBDs of OmpR (1OPC) [9], PhoB (1GXQ), and PhoB bound to DNA (1GXP) [42] revealed similar overall structures with Cα root mean square deviations (rmsd) from 1.38 to 1.71 Å. No large structural changes (Cα rmsd 1.15 Å) were reported for PhoB in free and bound conformations [42]. Likewise, the α-helical and β-stand elements of KdpE_DBD_ superpose well onto PhoB in DNA bound conformation with deviations restricted to loops connecting α7-α8 and α8 to the β-hairpin structure (Fig. 2C; the DNA corresponds to the PhoB recognition sequence). Generally, multiple contacts characterize DNA-protein interactions, [43], [44] which typically involve 24 amino acids residues and 12 nucleotides per protein dimer binding to two half-sites [45]. PhoB-DNA interactions were characterized by a larger number of contacts with the sugar-phosphate backbone and four base specific interactions involving residues T194, V197, R201 and R219 [42]. Although, KdpE does not contain identical residues (it is Y191, I194, H198 and T217 respectively at positions equivalent to those of PhoB shown in the previous line), the properties of three side chains are similar except for KdpE T217 and PhoB R219. However, the superposed structures reveal KdpE H198 is less-likely to participate in base specific interactions due to its shorter side-chain length when compared to R201 of PhoB (Fig. 2C). In the superposed structure, the steric clashes between DNA backbone and Y191 of KdpE indicates that the α8 recognition helix may have a different tilt angle with respect to the major groove to accommodate the larger side chain of KdpE Y191 (as compared to T194 of PhoB). These differences combined with variation in recognition sequences (TGTCA(T/C) and TTTA(T/C)A for PhoB and KdpE respectively) suggest that base-specific recognition may be different for the two proteins. Analysis KdpE_DBD_ structure superposed on PhoB-DNA complex revealed: (i) R193, conserved only among KdpE orthologs (Fig. 2B lower panel) had the potential to form base specific contacts (Fig. 2C) and (ii) residues conserved in OmpR/PhoB family namely R200 and T214 of KdpE superpose well onto R203 and T217 of PhoB that form a hydrogen bond (R203 NH1 T217OG1 in PhoB) in addition to salt bridges to the backbone in PhoB-DNA structure. The interactions between R203 and T217 of PhoB with each other and to DNA position the wing region of wHTH motif into the minor groove [42]. Therefore, residues R193, R200 and T214 of KdpE were mutated and the ability to promote gene expression *in vivo* and DNA binding was analyzed.

Although the key role played by major groove interactions is well established, a notable recent discovery is the important role of arginine residues in DNA minor groove interactions in a variety of protein-DNA complexes [46]. Arginine interactions occur at higher frequencies in narrow minor grooves (width <5 Å compared to 5.8 Å for ideal B-DNA) [46], which are formed by AT-rich sequences that are susceptible to DNA bending [47], [48]. Interaction between the R219 residue and an AT-rich minor groove was reported previously in the crystal structure of the DBD of PhoB with *pho* box DNA (Fig. 2C, R219 is shown in stick representation) [42]. Here, R219 penetrates the compressed minor groove to interact with T and A bases and the sugar backbones and leads to a 40° smooth bend in DNA. Such an arginine residue is conserved at structurally equivalent positions in many members of the OmpR/PhoB family, with the exception of KdpE, OmpR, and DrrB (Fig. S2). The corresponding residue in KdpE is the β-branched residue T217, the branch point sits close to the peptide backbone, which makes it unlikely to penetrate the minor groove formed by T-rich sequences between the S1 and S2 half-sites of kdpFABC_BS_. This suggests that the details of the interactions of KdpE_DBD_ with its cognate DNA will likely differ from that observed for PhoB. Because a crystal structure of DNA-KdpE_DBD_ complex would have provided detailed maps of the interactions, attempts were made to obtain co-crystals, which failed despite considerable efforts. DNA-protein complexes of members of the OmpR/PhoB family appear to be refractory to crystallization with the sole exception of DNA binding domain of PhoB [42]. In addition, determination of the structure of full-length RR from any response regulator family complexed to DNA continues to be a challenge.

### Effects of mutation of conserved residues in the wHTH motif of KdpE

**Figure 3:**
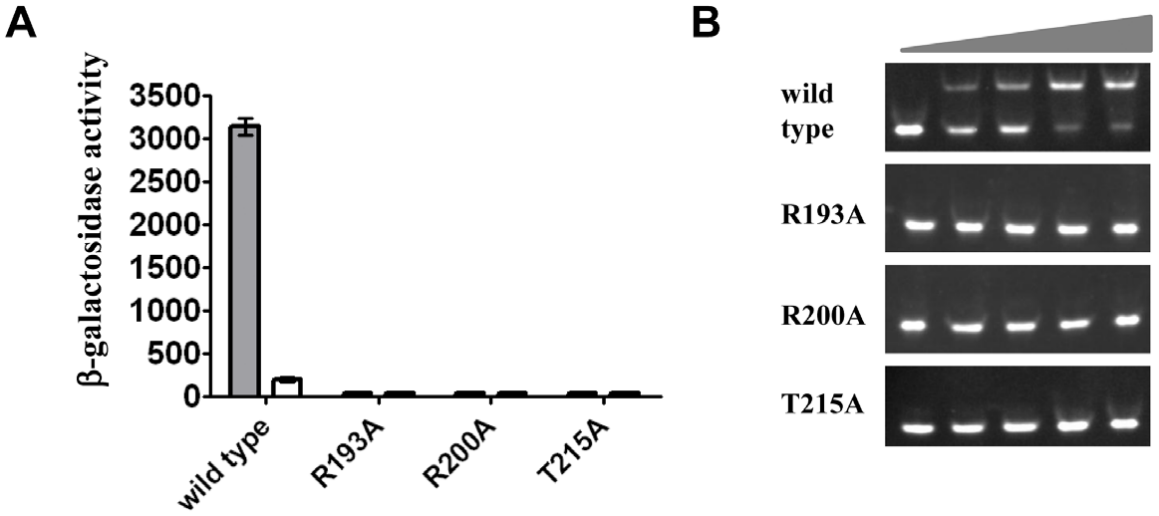
Effects of mutation of residues conserved in *kdpE_DBD_*. **A.** Comparison of β-galactosidase activities of KdpE mutants and wild-type KdpE in the *kdpFABC_Pro_-lacZ* fusion strain HAK003. Residues located in the α-8 (R193 and R200) and β-hairpin (T215) of KdpE (see Fig. 2) were targeted for mutagenesis to alanine. β-galactosidase (a reporter for *kdpFABC* expression) was measured in cells grown in media containing either K10 (white bar, 10 mM K^+^) or K0 (gray bar, 0 mM K^+^). **B.** EMSA showing effects of mutations in KdpE on interaction with the 30 bp DNA fragment representing its binding site. The triangles represent increasing molar ratios of 1∶0, 1∶1, 1∶2, 1∶4, and 1∶8 of DNA to purified mutants as indicated and wild-type KdpE_DBD_.

In contrast to wild-type KdpE, the three mutants (R193A, R200A and T215A) tested in the context of the full-length KdpE protein were incapable of responding to stress induced by changing the K^+^ concentration from 10 mM to 0 mM (Fig. 3A). This lack of response was investigated by purifying and assaying them for DNA binding. Mutant KdpE_DBD_ were incapable of binding to DNA in EMSA (Fig. 3B), even at a 1∶8 molar ratio of DNA to protein, which underscores the importance of these residues in stabilizing DNA-protein interactions. In the PhoB-DNA complex structure, R203, the residue equivalent to R200 of KdpE forms a salt bridge with O1P on the backbone of nucleotide T14 [42]. Mutations to the corresponding OmpR residue (R209) responsible for DNA backbone interactions impair its ability to stimulate expression of reporter genes fused to ssrA, ompF, and ompC promoters [28]. These data provide a plausible rationale for the disruption of the KdpE_DBD_R200A—*kdpFABC*_BS_ interaction. For T215 of KdpE, variants with mutations at equivalent residues in OmpR and PhoB are also defective in DNA binding due to loss of H-bonding with the DNA backbone [28], [49]. The inability of mutant KdpE R193A to bind to DNA is interesting because in most other family members the equivalent residue is of opposite charge as observed in PhoB (D196) and OmpR (D202) (Fig. S2). D196 of PhoB does not contribute to DNA interactions [42], however studies on PhoP from *M. tuberculosis* an ortholog of PhoB suggest a potential role for the equivalent residue E215 in base-specific interactions [50]. Because R193 is conserved among the KdpE family, and when mutated abrogates DNA interaction, it may play a role in base-specific recognition as suggested by the position in the superposition (Fig. 2C). Alternatively, the phenotype of KdpE R193A may be due to allosteric effects that alter DNA binding indirectly.

### Identifying the binding sites for KdpE in kdpFABC_BS_ DNA

**Figure 4:**
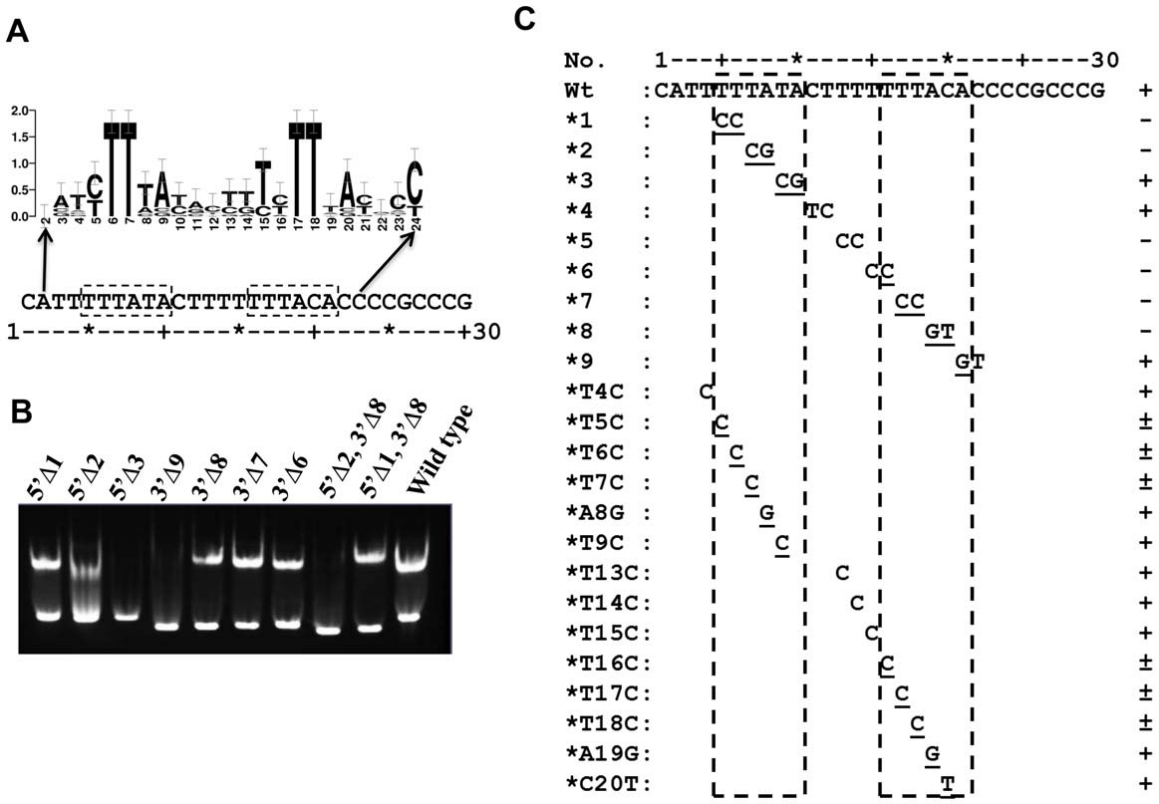
Identification and characterization of half-sites S1 and S2 on DNA that interacts with KdpE_DBD_. **A.** Sequence logo representation to highlight conserved sequences in a 24 bp stretch of *kdpFABC_BS_*. In the logo, the height of the letter represents its frequency of occurrence in a multiple sequence alignment (Fig. S3) and the error bars indicate the sampling error at individual positions. Two 6 bp imperfect direct repeats (TTTATA and TTTACA) separated by a 5 bp sequence are shown in dashed boxes below the logo. **B.** Identification of the minimal length of DNA required for binding KdpE. For EMSA, double-stranded DNA molecules with progressive deletions (indicated by Δ) at either 5′, 3′, or both ends were used (the nomenclature for oligonucleotides: 5′Δ2, 3′Δ8 (Fig. 4B, lane 9) refers to deletion of 2 and 8 bp from the 5′ and 3′ ends respectively of the wild-type (30 bp) DNA molecule; oligonucleotides used are shown in Table S2). The interpretation of EMSA was qualitative: discreet band shifts as observed in Fig. 4B, lane 1 were considered a positive reaction (+), whereas no shift (Fig. 4B, lane 3) was scored negative (−) and smeared bands as exemplified by Fig. 4B, lane 2 were considered partial binding. **C.** Effects of changes in DNA sequence on the KdpE_DBD_-DNA interaction. A summary of EMSA data (data not shown) using the 30 bp *kdpFABC_BS_* sequence and modified oligonucleotides (only specific two or one nucleotide substitutions are noted) are presented. The scoring of EMSA analysis was as described above. The dashed boxes represent the 6 bp direct repeats that form half-sites S1 and S2.

To identify KdpE binding half-sites and their specific sequences, a multiple sequence alignment of regions upstream of the *kdpFABC* operon from a variety of bacteria was generated (Fig. S3). Figure 4A shows a logo representation of sequence conservation. The analysis revealed the presence of two 6 bp direct repeats with 1 base variation (labeled half-sites S1 and S2) that are separated by AT-rich 5 bp sequence. The half-sites are also AT rich, and within each half-site the first two bases are deoxythymidines, which are invariant across various genera (Fig. 4A). We propose that KdpE_DBD_ binds to half-sites S1 and S2. To define the minimal binding region, a series of DNA molecules progressively missing nucleotides at the 5′ and 3′ ends of S1 and S2, respectively, were generated. EMSA analyses of KdpE_DBD_ with truncated DNA molecules showed that a 21 bp fragment with only 3 bp beyond the 5′ end of S1 and only 1 bp beyond the 3′ end of S2 is sufficient for binding to KdpE (Fig. 4B). These results concur with footprinting analysis identifying a 23 bp KdpE binding element situated between −72 and −50 of the transcription start site for the *kdpFABC* operon [24].

To define specificity, DNA bases critical to KdpE_DBD_ binding were identified by scanning an 18 bp stretch by introducing dinucleotide changes (Fig. 4C). In all cases, base changes were transitions. Modified DNA molecules at a 1∶8 molar ratio of DNA to protein were scored as positive, negative, or weakly positive for binding based on mobility shifts. Mutations in the first four bases (TTTA) that are same in each of the half-sites abolished DNA interactions. A subsequent fine-grain analysis using single base substitutions showed that partial binding of KdpE_DBD_ to DNA occurred for all single base substitutions, indicating that more than one base must be changed to abolish binding.

### Quantitative analysis of the KdpE_DBD_ interaction with DNA

**Figure 5:**
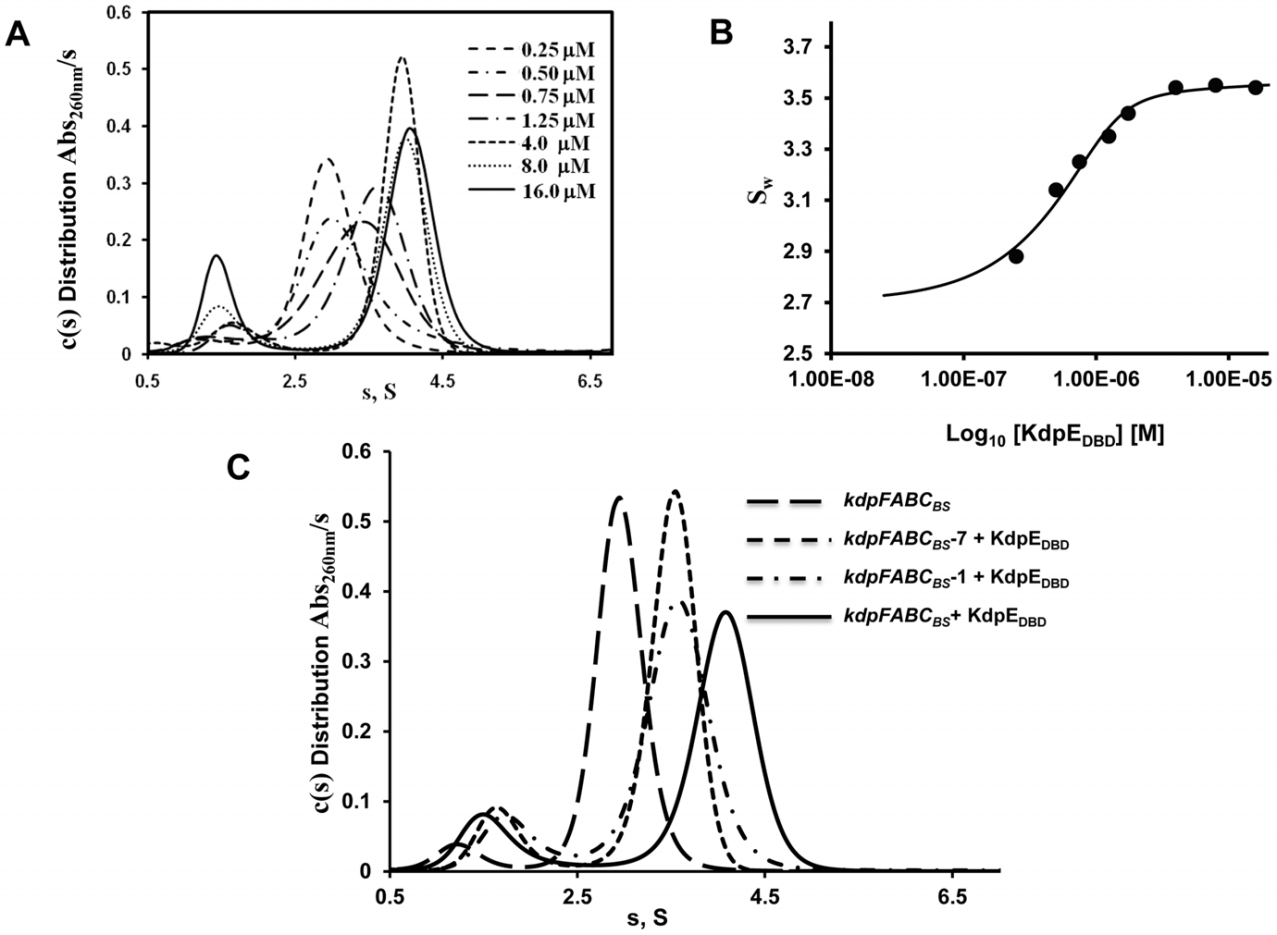
Sedimentation velocity analysis of KdpE_DBD_—*kdpFABC_BS_* association. **A.** Continuous distribution of sedimentation coefficients [c(s)] as a function of increasing concentration of protein against a fixed concentration of *kdpFABC_BS_* DNA (0.5 µM). The protein concentrations used varied between 0.25 and 16 µM as shown. The largest complex with sedimentation coefficient of 4.1 S was observed at protein concentration of 4 to 16 µM. Independent experiments established the sedimentation coefficients of KdpE_DBD_ and *kdpFABC_BS_* at 1.4 S and 2.8 S respectively (data not shown). **B.** A plot of the weight average sedimentation coefficients (S_w_) against the concentration of KdpE_DBD_ is shown. Analysis of the isotherm indicated that DNA was saturated beginning at 8-fold molar excess of KdpE_DBD_ protein. **C.** SV c(s) distributions comparing binding of KdpE_DBD_ to the S1 and S2 sites individually and to the both sites simultaneously. Wild-type DNA with both sites intact (*kdpFABC_BS_*), functional S1 (*kdpFABC_BS_ —*7) and S2 (*kdpFABC_BS_ —*1) sites were analyzed with a 16-fold molar excess of Kdp_DBD_. Complexes with DNA possessing single sites have sedimentation coefficients of 3.5 S whereas when both sites were occupied a 4.1 S species was formed.

In Figure 5A, the peak corresponding to the 30 bp DNA at the sedimentation coefficient of 2.8 S shifted to 4.1 S with increasing concentrations of KdpE_DBD_. The formation of the complex represented a fast equilibrium process (k_off_>10^−2^/sec on the time scale of sedimentation [51]), as indicated by the change in s-value of the KdpE_DBD_—*kdpFABC_BS_* complex as a function of increasing KdpE_DBD_ protein concentration. To evaluate saturation of binding sites, an isotherm analysis [52] was conducted by integrating the entire c(s) distribution to generate a weight-averaged s-value (S_w_) and plotting it against the concentration of KdpE_DBD_. The S_w_ did not change beyond the 8-fold molar excess of KdpE_DBD_, which confirms full complex formation (Fig. 5B). Because the entire c(s) distribution (as in Fig. 5A) was integrated the reported maximal S_w_ value of 3.5 S (Fig. 5B) is less than the true value of 4.1 S due to effect of the smaller s-value of the excess unbound species on the larger fully complexed species [53]. As shown in Fig. 5C, the maximal s-value of the complexed species was 4.1 S. Furthermore, the calculated mass from SV data shows formation of 2∶1 protein to DNA complex (Table S4) and when fitted to a two site model, the isotherm binding curve gave *K_d_*s of 90 nM and 300 nM.

**Figure 6:**
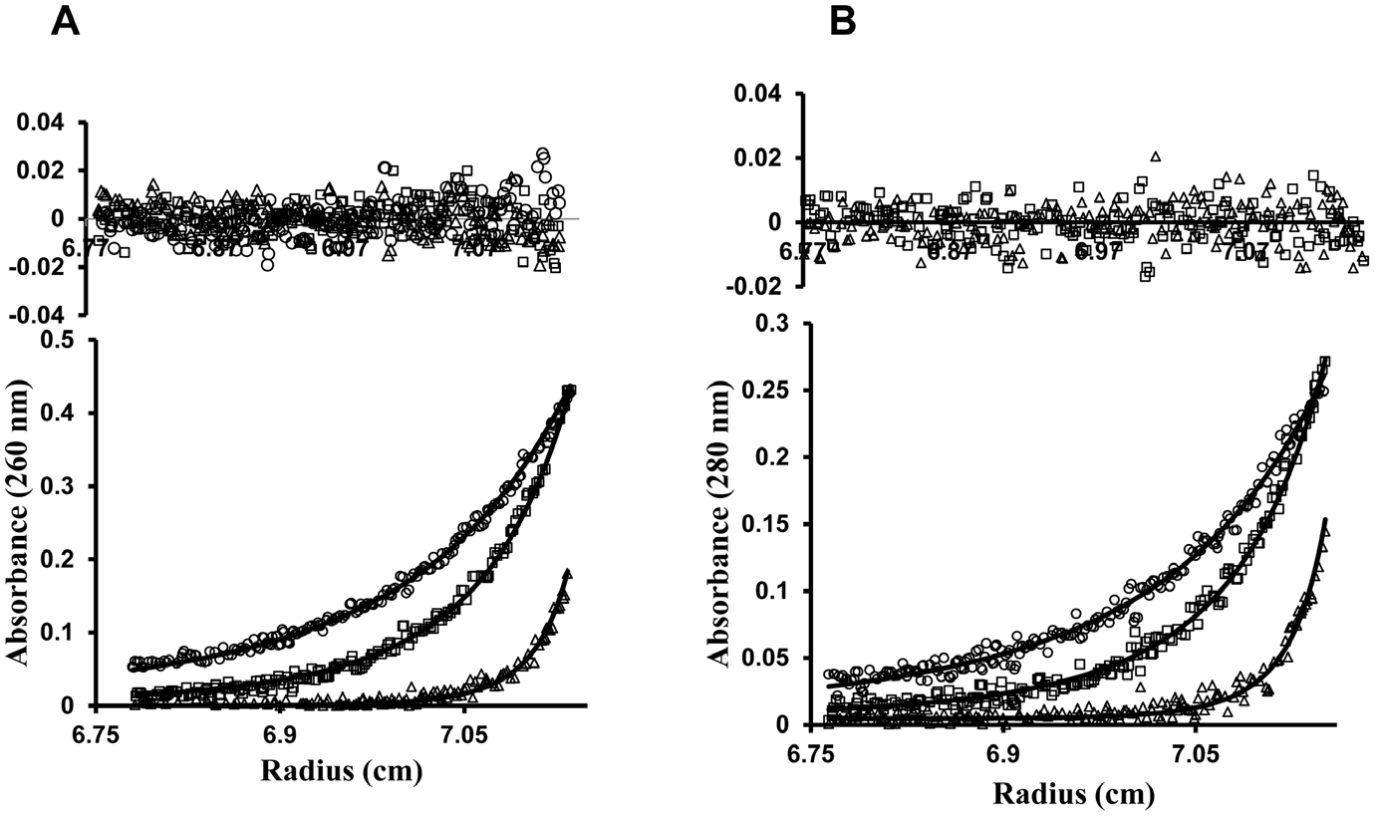
Sedimentation equilibrium analysis of the KdpE_DBD_—*kdpFABC_BS_* complex to determine the *K_d_* and stoichiometry. Representative SE profiles of 0.63 µM *kdpFABC_BS_* and 2.5 µM KdpE_DBD_ generated from data collected at 260 nm (A) and 280 nm (B) are shown. Mixtures of KdpE_DBD_ and *kdpFABCBS* were spun at 9,000 (•), 19,800 (□) and 34,000 (Δ) rpm. The data were fit to a two site binding model with symmetrical sites using SEDPHAT. The root mean square deviation values for the fits were 0.0039 and 0.0034 for samples at 260 and 280 nm, respectively. The residuals (inset) showed no systematic deviations. The fitted values are shown in Tables 2 and 3.

The estimated weight of the KdpE_DBD_:DNA complex from the SV experiments was 47500 Da indicating a 2∶1 complex, but this estimation can be impeded by the shape and hydration of the complex (Table S4) [36]. Since the SE is uninfluenced by shape, and the contributions to absorbance at 260 nm from KdpE_DBD_ was low and therefore could be ignored, we calculated the molecular weight of the complex as a single species as described by Kar et al., [36]. The calculated molecular weight of 43000±2000 Da from SE analysis indicates a 2∶1 stoichiometry of KdpE_DBD_ to DNA (Table 2, Fig. 6), is in overall agreement with the conclusion from SV experiments. Using the binding stoichiometry KdpE_DBD_: DNA of 2∶1, the experimental data was then fitted to a two to one model in SEDPHAT namely, A+B+B→AB+B→ABB revealing upper limits for the dissociation constants (*K_d_*) of 80±25 nM and 300±90 nM for the interaction of KdpE_DBD_ with *kdpFABC_BS_* [38]. Here the model does not discriminate between the two sites and assumes that first an AB complex is formed which subsequently binds to a second molecule [37]. The initial complex could form at either subsites S1 or S2, and then a second molecule of protein binds to the available adjacent site. Because of the nature of the model, the equation will enforce a four-fold difference between the *K_d_*s at the two sites when one considers two hypothetical sites that absolutely equal in all respects [37], [54]. When the macroscopic cooperativity factor in SEDPHAT was analyzed to evaluate the possibility of cooperativity, no significant deviation from the global reduced critical χ^2^ was observed. This suggests that the two binding sites likely are equivalent and independent within the limits of the data.

**Table 2:** Comparison of molecular masses calculated from sequence and sedimentation equilibrium analysis of KdpE_DBD_, its DNA recognition sequence, and their complexes.

Sample	Theoretical Mass (Da)[Protein: DNA]	Calculated Mass (Da)Sedimentation equilibrium
KdpE_DBD_	12022	11200±500
*kdpFABC_BS_*	18410	20000±1500
*kdpFABC_BS_—7*	18412	–
*kdpFABC_BS_—1*	18412	–
KdpE_DBD_+*kdpFABC_BS_*	30824 [1∶1]/42454 [2∶1]	43000±2000
KdpE_DBD_+*kdpFABC_BS_—7*	30434 [1∶1]/42456 [2∶1]	30000±2500
KdpE_DBD_+*kdpFABC_BS_—1*	30434 [1∶1]/42456 [2∶1]	30000±1500

*kdpFABC_BS_* represents the wild-type DNA sequence, whereas *kdpFABC_BS_—1* and *kdpFABC_BS_—7* DNA have mutations that abolish binding at half-sites S1 and S2, respectively. All DNAs are 30 bp in length.

**Figure 7:**
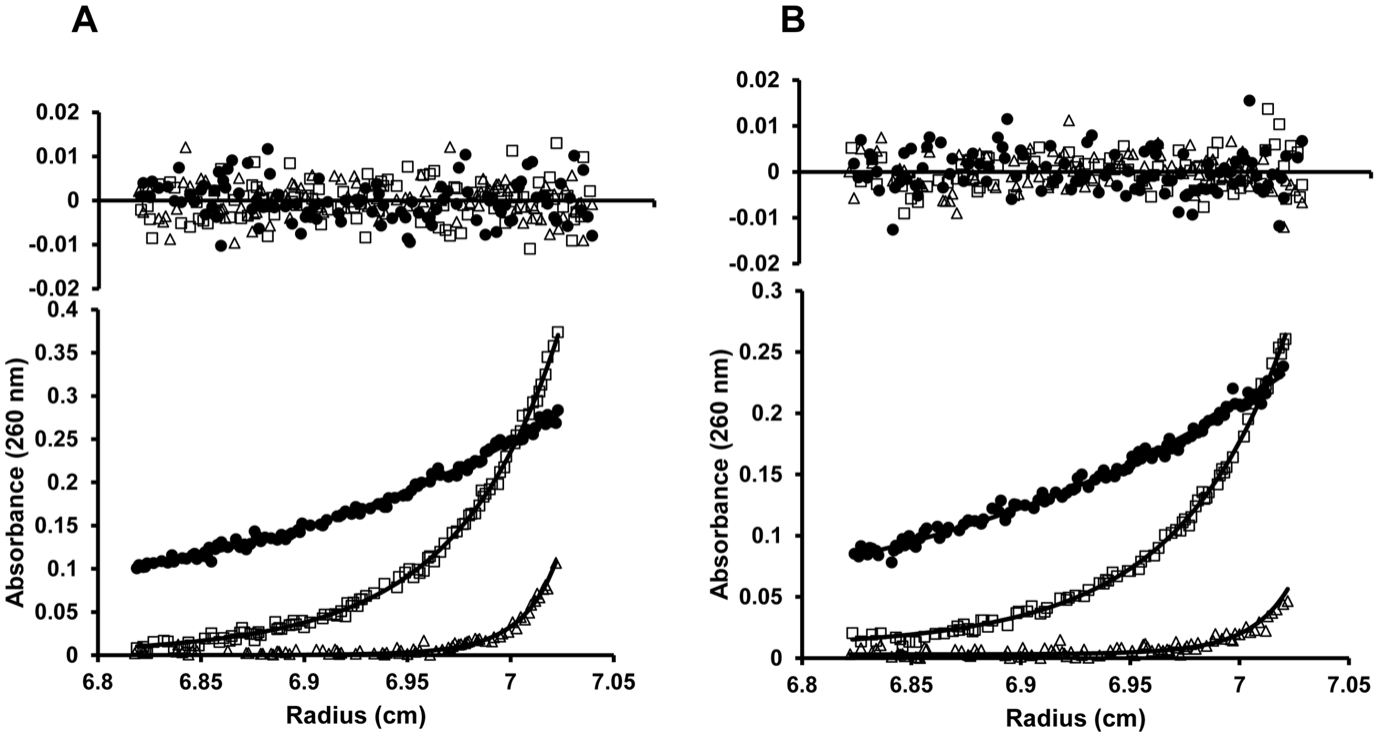
*Binding analysis of the half-sites of kdpFABC_BS_*. SE analysis of binding of KdpE_DBD_ to S1 (*kdpFABC_BS_—7*) (**A**)and S2 (*kdpFABC_BS_*—*1*) (**B**) half-sites revealed a 1∶1 stoichiometry. Mixtures of KdpE_DBD_ and DNA were spun at 9,000 (•), 19,800 (□) and 34,000 (Δ) rpm. The *K_d_*s obtained for KdpE_DBD_ binding at half-sites S1 was 350±100 nM and for S2 was 200±100 nM using a one site binding model (AB) in SEDPHAT. The molecular weights calculated from the SE data were 30,000±1,500 for *kdpFABC_BS_—1* and 30,000±2,500 for *kdpFABC_BS_—7*.

The *K_d_*s obtained from the S_w_ isotherm binding and SE analyses were in agreement, indicating that KdpE_DBD_ interacts strongly with *kdpFABC_BS_*. However, the *K_d_* values determined cannot be specifically assigned to either half-site. To examine the interaction of KdpE_DBD_ with individual half-sites and to test if cooperativity plays a significant role in binding, 30 bp DNA molecules with mutations in S1 and S2 half-sites were used. The DNAs containing double nucleotide substitutions failed to interact with KdpE_DBD_ (Fig. 4C) due to weaker binding and/or changes in stoichiometry. This dichotomy was not resolvable by the EMSA assay due to its inherent limitations as a non-equilibrium method [55]. Therefore, SV experiments were conducted using the modified sequences *kdpFABC_BS_*—*1* and *kdpFABC_BS_*—*7* (these have mutations in S1 and S2 half-sites respectively that abrogate KdpE_DBD_ binding) in the presence of excess KdpE_DBD_ (Fig. 5C). Both mutant DNA-KdpE_DBD_ complexes sedimented as a 3.5 S species, and such values were significantly lower than that of the wild-type *kdpFABC_BS_*— KdpE_DBD_ complex (4.1 S) suggestive of altered stoichiometry. To confirm that the 3.5 S species were indicative of a different binding stoichiometry, SE analyses were conducted. The results indicated that the 3.5 S species were in a 1∶1 stoichiometry (Fig. 7; Table 3). Additionally, the dissociation constants calculated from SE data were similar, with *K_d_*s of 350±100 and 200±100 nM for half-sites S1 and S2, respectively (Table 2). The change between the *K_d_*s at S1 and S2 was small (less than two-fold) suggesting the binding at these sites are not very different. The small differences in binding affinity are unsurprising because of the binding sites (TTTATA and TTTACA for sites S1 and S2 respectively) are nearly identical with one base change at a position that has been shown to have no effect on interaction with KdpE_DBD_ (Fig. 4C).

**Table 3:** Binding affinities of KdpE_DBD_ to wild-type and mutant DNA molecules determined by Sedimentation Equilibrium analysis.

Species	*K_d1_* (nM)	*K_d2_* (nM)
KdpE_DBD_+*kdpFABC_BS_—1* a	-	200±100 (S2)
KdpE_DBD_+*kdpFABC_BS_—7* a	350±100 (S1)	-

a The apparent *K_d_* values assigned to S1 and S2 are based on values obtained using *kdpFABC_BS_-1*and *kdpFABC_BS_-7* that have single functional binding sites at S2 and S1 respectively. Error limits were generated using F-statistics with a confidence interval of 1σ.

For members of the OmpR/PhoB family the few analyses of DNA-protein interactions under equilibrium conditions available describe binding to a pair of half-sites that form a single recognition site. Fluorescence anisotropic monitoring of protein-DNA interactions showed that the DBD of PhoB binds *pho* box DNA containing two half-sites with a 7-fold higher affinity (63 nM) than the non-phosphorylated, full-length PhoB (440 nM) [40], whereas upon phosphorylation the affinity increases to 9.7 nM [56]. However, little is known about binding at half-sites of the *phoB* box. In case of OmpR, the isolated DBD (OmpRc) binds weakly to recognition sites F1 and C1 at the *ompR* and *ompC* promoters respectively [57], [58]. Based on perturbations of resonances in NMR analysis, Rhee et al., [28] concluded that OmpRc binds to isolated half-sites C1b and C1a within the C1 recognition site. The proposed stronger binding at the 3′ half-site C1b was based on greater chemical shift perturbations over the C1a site which led to a model of sequential loading of OmpR first at C1b followed by C1a [28]. A comparable overall conclusion of sequential loading at half-sites of F1a and F1b of F1 promoter by phosphorylated form of full-length OmpR was reached by Inouye and colleagues using EMSA with F1b site binding more tightly than F1a [27]. They invoked cooperative binding mediated by receiver domains to explain the requirement for binding at both half-sites to form a stable complex [27]. However, these are largely qualitative descriptions of protein-DNA interactions. In contrast, our quantitative equilibrium binding analysis of KdpE_DBD_ to its half-sites led to three salient observations: (i) The binding of KdpE_DBD_ at each half-site was moderately strong (*K_d_* in the range of 200 to 350 nM); (ii) The equilibrium dissociation constants of the two-half sites are comparable; and (iii) The inactivation of one half-site does not affect the affinity of KdpE_DBD_ for the other. These results indicate that KdpE_DBD_ binds independently to the S1 and S2 half-sites of *kdpFABC_BS_* with equivalent affinity but without significant cooperativity.

In conclusion, our studies identified residues of KdpE_DBD_ that participate in DNA binding, the location of the half-sites on the DNA; and the nucleotide bases essential for protein binding. Although the structure of KdpE_DBD_ is similar to that of other members of the OmpR/PhoB family of proteins, several important differences exist. KdpE_DBD_ lacks a conserved arginine residue in the β-hairpin of the wHTH motif that interacts with the DNA minor groove as observed in PhoB. Unlike OmpR, the DBDs of both PhoB and KdpE bind their cognate DNA with moderate affinities (range of *K_d_*s 100–400 nM); however, only the DBD of PhoB can initiate transcription [40]. The KdpE_DBD_-DNA binding at each half site is independent and equivalent and therefore unlikely to involve the hierarchical loading observed in other systems. Similar information is available for one member each of the NarL/FixJ and LytR/AgrA families of RR [59]. For TodT in the NarL/FixJ group, binding to half-sites is characterized by low affinities (micromolar range) and weak cooperativity [60]. Kinetic analysis of binding by the RRs PlnC and PlnD of the LytR/AgrA families to the P*_plnA_* recognition sequence showed significantly higher affinity to the right (3′) half-site than the left half-site, and binding to the latter half-site was cooperatively dependent on the former [59]. To our knowledge, ours is the first report of equilibrium binding analysis at half-sites of a recognition site for a member of the OmpR/PhoB family, the largest group among all RRs. Further studies are necessary to identify the role of phosphorylation of the receiver domain in either enhancing the affinity of binding to DNA and/or in cooperative interactions at the two half-sites of the KdpE recognition site.

### Data Bank Accession Codes

The atomic coordinates for KdpE_DBD_ have been deposited in Protein Data bank (accession number 3zq7).

## Supporting Information

Figure S1 **Purification and characterization of KdpE_DBD_ and its mutants.** Size exclusion chromatographic analyses and SDS-PAGE (inset) of purified KdpE_DBD_ showed a single peak and band respectively.
(TIF)

Figure S2 Multiple sequence alignment of amino acid sequences of members of the OmpR/PhoB family. The abbreviations used correspond to the PDB accession code followed by the four letter name of the protein. The numbers reflect the residue number of the full-length protein. Only the winged helix-turn-helix (wHTH) motif sequences derived from proteins with known 3D-structures is represented in the alignment prepared using Tcoffee server (http://tcoffee.vital-it.ch/cgi-bin/Tcoffee/tcoffee_cgi/index.cgi) and shaded using the program Boxshade (fraction of sequences that must agree for shading = 0.8). Residues in KdpE targeted for mutagenesis namely R193, R200 and T215 are indicated by stars, whereas the+sign points to R219 of PhoB that interacts with the minor groove of DNA.
(TIF)

Figure S3 Multiple sequence alignment of DNA sequence regions of the promoter region of *kdpFABC* operon. The alignment was prepared using CLUSTALW in slow mode and shaded with Boxshade (fraction of sequences that must agree for shading = 0.8). The abbreviations used were: E_coli, *Escherichia coli*; S_typhi, *Salmonella typhimurium*; P_fluor, *Pseudomanas fluorscens*; R_palus, *Rhodobacter palustris*; S_aureus, *Stapholoccus aureus*; E_faeca, *Enterococcus faecalis*; M_tuber, *Mycobacterium tuberculosis*.
(TIF)

Table S1 **Primers used for cloning KdpE_DBD_, full-length KdpE and point mutants.**
(DOC)

Table S2 **Primers for EMSA.** Sequences of one of the two strands in a double-stranded DNA molecule are shown. Changes in sequence when compared to wild-type *kdpFABC_BS_* are underlined and the Δ indicates deletions at the 5′ and 3′ ends of DNA molecules.
(DOC)

Table S3
**Parameters used in sedimentation velocity and sedimentation equilibrium analyses.** The partial specific volume (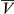) for *kdpFABC_BS_* DNA and its mutated versions were calculated from GC content.^54^ The GC content for the DNA used in these experiments was ∼40%. The 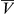 of 0.590 cm^3^ g^−1^ was used for the three DNA molecules. The vbars assume no significant change in volume upon the protein DNA interaction.(DOC)

Table S4 **Molecular masses estimated from sedimentation velocity experiments.** For the complex, the KdpE_DBD_ concentration was 10 µM and the DNA concentration held constant at 0.63 µM.
(DOC)
