# Supplementary figures and images for: Structure-Function Studies of DNA Binding Domain of Response Regulator KdpE Reveals Equal Affinity Interactions at DNA Half-Sites
Source: PLoS One. 2012 Jan 23;7(1):e30102. doi: 10.1371/journal.pone.0030102 (PMC3264566; doi:10.1371/journal.pone.0030102)

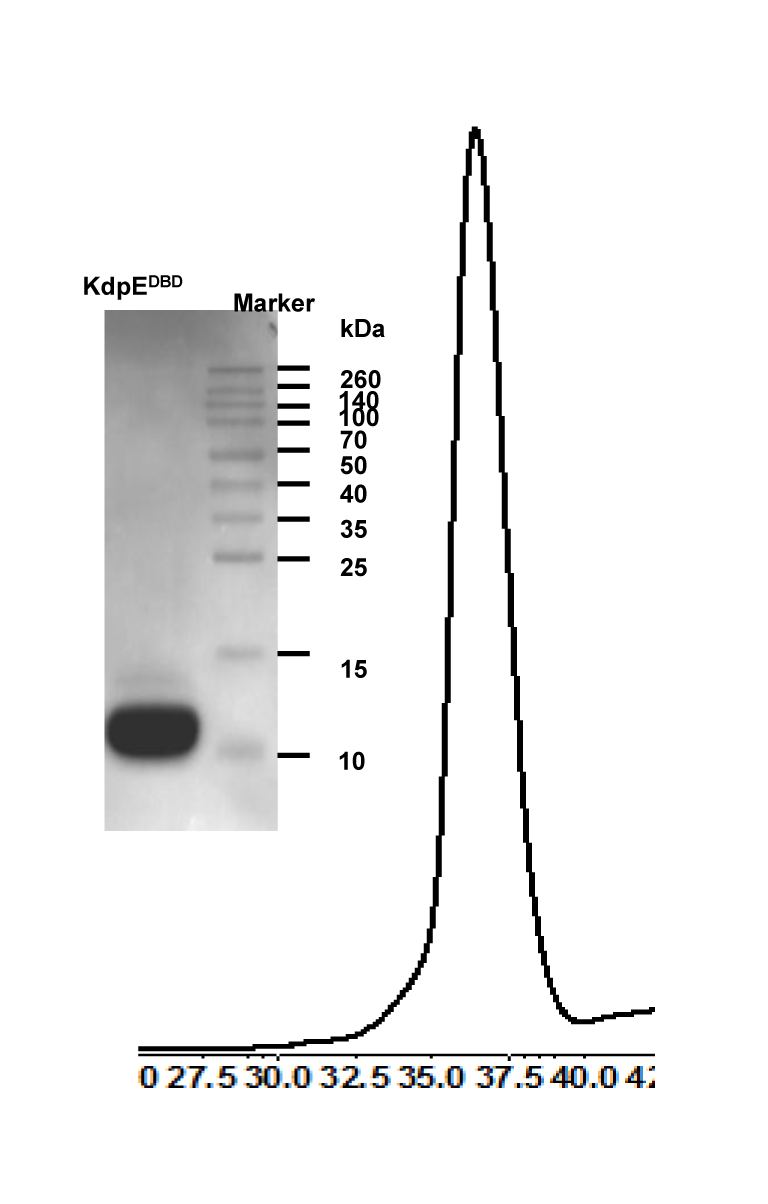

Supplement: Figure S1 — Purification and characterization of KdpEDBD and its mutants. Size exclusion chromatographic analyses and SDS-PAGE (inset) of purified KdpEDBD showed a single peak and band respectively. (TIF) [file pone.0030102.s001.tif]

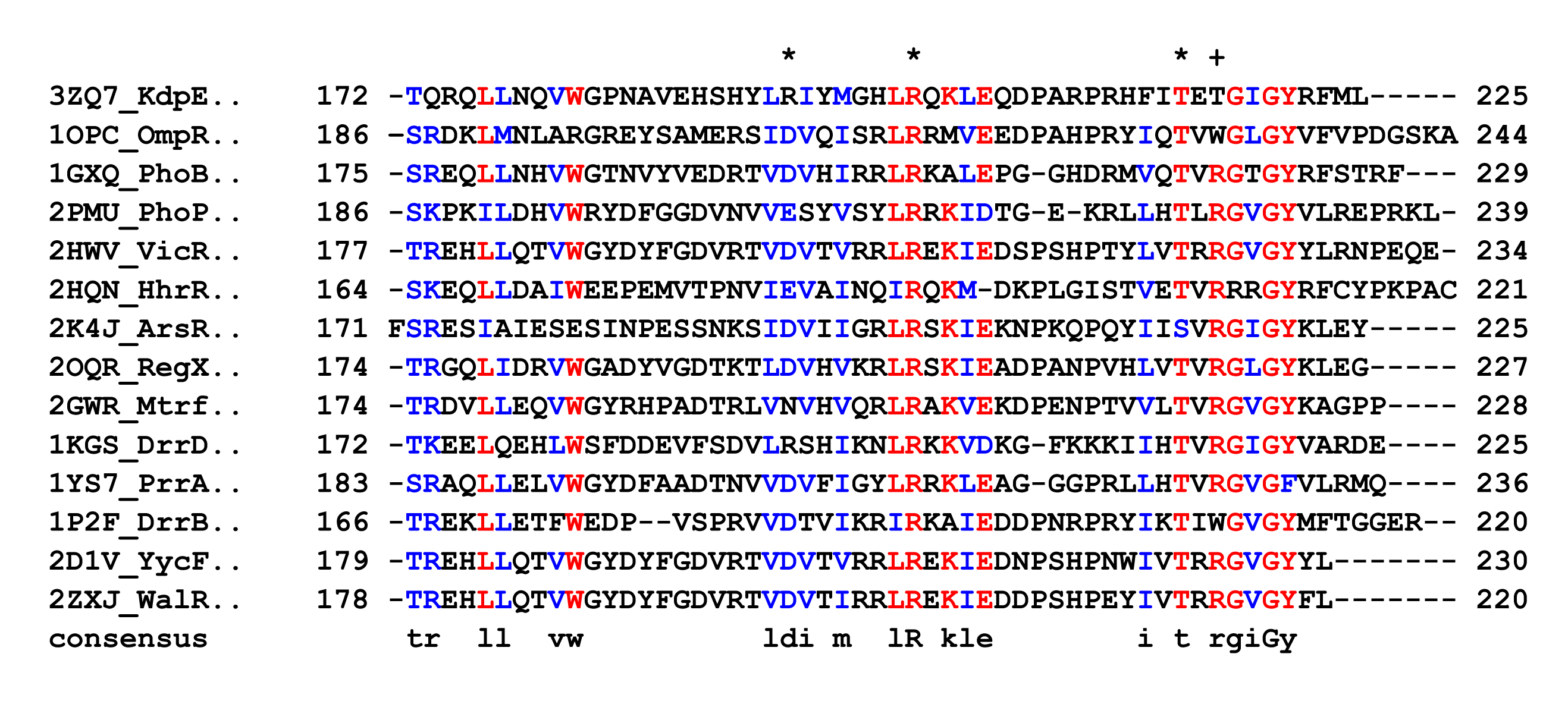

Supplement: Figure S2 — Multiple sequence alignment of amino acid sequences of members of the OmpR/PhoB family. The abbreviations used correspond to the PDB accession code followed by the four letter name of the protein. The numbers reflect the residue number of the full-length protein. Only the winged helix-turn-helix (wHTH) motif sequences derived from proteins with known 3D-structures is represented in the alignment prepared using Tcoffee server (http://tcoffee.vital-it.ch/cgi-bin/Tcoffee/tcoffee_cgi/index.cgi) and shaded using the program Boxshade (fraction of sequences that must agree for shading = 0.8). Residues in KdpE targeted for mutagenesis namely R193, R200 and T215 are indicated by stars, whereas the+sign points to R219 of PhoB that interacts with the minor groove of DNA. (TIF) [file pone.0030102.s002.tif]

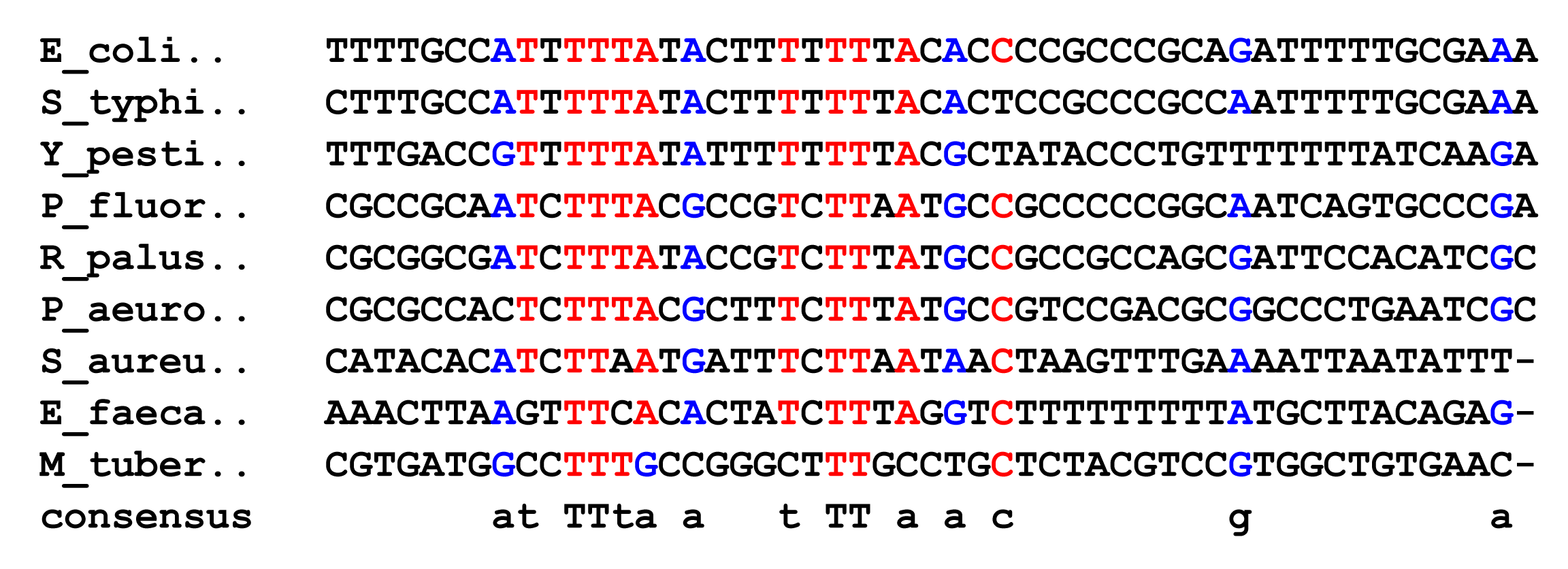

Supplement: Figure S3 — Multiple sequence alignment of DNA sequence regions of the promoter region of kdpFABC operon. The alignment was prepared using CLUSTALW in slow mode and shaded with Boxshade (fraction of sequences that must agree for shading = 0.8). The abbreviations used were: E_coli, Escherichia coli; S_typhi, Salmonella typhimurium; P_fluor, Pseudomanas fluorscens; R_palus, Rhodobacter palustris; S_aureus, Stapholoccus aureus; E_faeca, Enterococcus faecalis; M_tuber, Mycobacterium tuberculosis. (TIF) [file pone.0030102.s003.tif]
