# Supplementary material for: Structure-Function Studies of DNA Binding Domain of Response Regulator KdpE Reveals Equal Affinity Interactions at DNA Half-Sites
Source: PLoS One. 2012 Jan 23;7(1):e30102. doi: 10.1371/journal.pone.0030102 (PMC3264566; doi:10.1371/journal.pone.0030102)
Supplement: Table S1 — Primers used for cloning KdpEDBD, full-length KdpE and point mutants. (DOC) [file pone.0030102.s004.doc]

| **Primers for cloning *kdpE_DBD_* and full-length KdpE** (Fp and Rp denote forward and reverse primers, respectively; for cloning KdpE, DBD-HindIII-Rp was used with FL-NcoI-Fp whereas *kdpE_DBD_* was cloned using DBD-HindIII-Rp and DBD-NcoI-Fp) | |
| --- | --- |
| DBD-NcoI-Fp | GCTCATCCATGGCAAACGTTCTGATTGTTGAAGATG |
| DBD-HindIII-Rp | ATCTGAAAGCTTTCAAAGCATAAACCGATAGCCAATACCG |
| FL-NcoI-Fp | GCTCATCCATGGCAAACGTTCTGATTGTTGAAGATG |
| **Primers for introducing mutations in *kdpE_DBD_* and KdpE** | |
| R193A-Fp | CACTATTTGGCAATTTATATG |
| R193A-Rp | CATATAAATTGCCAAATAGTG |
| R200A-Fp | GACATCTGGCACAAAAACTG |
| R200A-Rp | CAGTTTTTGTGCCAGATGTC |
| T215A-Fp | GCCATTTCATTGCTGAAACCG |
| T215A-Rp | CGGTTTCAGCAATGAAATGGC |
