# Supplementary material for: Structure-Function Studies of DNA Binding Domain of Response Regulator KdpE Reveals Equal Affinity Interactions at DNA Half-Sites
Source: PLoS One. 2012 Jan 23;7(1):e30102. doi: 10.1371/journal.pone.0030102 (PMC3264566; doi:10.1371/journal.pone.0030102)
Supplement: Table S2 — Primers for EMSA. Sequences of one of the two strands in a double-stranded DNA molecule are shown. Changes in sequence when compared to wild-type kdpFABCBS are underlined and the Δ indicates deletions at the 5′ and 3′ ends of DNA molecules. (DOC) [file pone.0030102.s005.doc]

| ompF_Pro_ | ATTTATTTGAAAAAAAGTATAAAAATG |
| --- | --- |
| *kdpFABC_BS_* | CATTTTTATACTTTTTTTACACCCCGCCCG |
| *kdpFABC_BS_*―5’Δ1 | ATTTTTATACTTTTTTTACACCCCGCCCG |
| *kdpFABC_BS_*―5’Δ2 | TTTTTATACTTTTTTTACACCCCGCCCG |
| *kdpFABC_BS_*―5’Δ3 | TTTTATACTTTTTTTACACCCCGCCCG |
| *kdpFABC_BS_*―3’Δ9 | CATTTTTATACTTTTTTTACA |
| *kdpFABC_BS_*―3’Δ8 | CATTTTTATACTTTTTTTACAC |
| *kdpFABC_BS_*―3’Δ7 | CATTTTTATACTTTTTTTACACC |
| *kdpFABC_BS_*―3’Δ6 | CATTTTTATACTTTTTTTACACCC |
| *kdpFABC_BS_*―3’Δ3 | CATTTTTATACTTTTTTTACACCCCGC |
| *kdpFABC_BS_*―5’∆2, 3’∆8 | TTTTTATACTTTTTTTACAC |
| *kdpFABC_BS_*―5’∆1, 3’∆8 | ATTTTTATACTTTTTTTACAC |
| *kdpFABC_BS_*―1 | CATTCCTATACTTTTTTTACACCCCGCCCG |
| *kdpFABC_BS_*―2 | CATTTTCGTACTTTTTTTACACCCCGCCCG |
| *kdpFABC_BS_*―3 | CATTTTTACGCTTTTTTTACACCCCGCCCG |
| *kdpFABC_BS_*―4 | CATTTTTATATCTTTTTTACACCCCGCCCG |
| *kdpFABC_BS_*―5 | CATTTTTATACTCCTTTTACACCCCGCCCG |
| *kdpFABC_BS_*―6 | CATTTTTATACTTTCCTTACACCCCGCCCG |
| *kdpFABC_BS_*―7 | CATTTTTATACTTTTTCCACACCCCGCCCG |
| *kdpFABC_BS_*―8 | CATTTTTATACTTTTTTTGTACCCCGCCCG |
| *kdpFABC_BS_*―9 | CATTTTTATACTTTTTTTACGTCCCGCCCG |
| *kdpFABC_BS_*―3TC | CACTTTTATACTTTTTTTACACCCCGCCCG |
| *kdpFABC_BS_*―4TC | CATCTTTATACTTTTTTTACACCCCGCCCG |
| *kdpFABC_BS_*―5TC | CATTCTTATACTTTTTTTACACCCCGCCCG |
| *kdpFABC_BS_*―6TC | CATTTCTATACTTTTTTTACACCCCGCCCG |
| *kdpFABC_BS_*―7TC | CATTTTCATACTTTTTTTACACCCCGCCCG |
| *kdpFABC_BS_*―8AG | CATTTTTGTACTTTTTTTACACCCCGCCCG |
| *kdpFABC_BS_*―13TC | CATTTTTATACTCTTTTTACACCCCGCCCG |
| *kdpFABC_BS_*―14TC | CATTTTTATACTTCTTTTACACCCCGCCCG |
| *kdpFABC_BS_*―15TC | CATTTTTATACTTTCTTTACACCCCGCCCG |
| *kdpFABC_BS_*―16TC | CATTTTTATACTTTTCTTACACCCCGCCCG |
| *kdpFABC_BS_*―17TC | CATTTTTATACTTTTTCTACACCCCGCCCG |
| *kdpFABC_BS_*―18TC | CATTTTTATACTTTTTTCACACCCCGCCCG |
| *kdpFABC_BS_*―19AG | CATTTTTATACTTTTTTTGCACCCCGCCCG |
| *kdpFABC_BS_*―20CT | CATTTTTATACTTTTTTTATACCCCGCCCG |
