# Supplementary material for: Structure-Function Studies of DNA Binding Domain of Response Regulator KdpE Reveals Equal Affinity Interactions at DNA Half-Sites
Source: PLoS One. 2012 Jan 23;7(1):e30102. doi: 10.1371/journal.pone.0030102 (PMC3264566; doi:10.1371/journal.pone.0030102)
Supplement: Table S3 — Parameters used in sedimentation velocity and sedimentation equilibrium analyses. The partial specific volume () for kdpFABCBS DNA and its mutated versions were calculated from GC content.54 The GC content for the DNA used in these experiments was ∼40%. The of 0.590 cm3 g−1 was used for the three DNA molecules. The vbars assume no significant change in volume upon the protein DNA interaction. (DOC) [file pone.0030102.s006.doc]

| **Species** | - **cm^3^g^-1a^** | **R^b^** | - **_complex_ cm^3^g^-1c^** |
| --- | --- | --- | --- |
| ***kdpFABC_BS_*** | 0.590^d^ | - | - |
| **KdpE_DBD_** | 0.7438 | [1:1] 0.64 / [2:1] 1.30 | [1:1] 0.65 / [2:1] 0.676 |

^a^ The
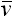
 of single species

^b^ R is the ratio of the mass of the protein to DNA

^c^ The
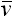
 of the protein:DNA complex at 1:1 and 2:1 ratios

^d^ The
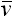
 of 0.590 cm^3^g^-1^ was also used for *kdpFABC_BS_―1* and *kdpFABC_BS_―7*
