# Supplementary material for: Structure-Function Studies of DNA Binding Domain of Response Regulator KdpE Reveals Equal Affinity Interactions at DNA Half-Sites
Source: PLoS One. 2012 Jan 23;7(1):e30102. doi: 10.1371/journal.pone.0030102 (PMC3264566; doi:10.1371/journal.pone.0030102)
Supplement: Table S4 — Molecular masses estimated from sedimentation velocity experiments. For the complex, the KdpEDBD concentration was 10 µM and the DNA concentration held constant at 0.63 µM. (DOC) [file pone.0030102.s007.doc]

| **Sample** | **Theoretical Mass (Da)**  **[Protein : DNA]** | **Calculated mass (Da) Sedimentation Velocity** | - **cm^3^g^-1 a^** |
| --- | --- | --- | --- |
| KdpE_DBD_ | 12022 | 12103 | 0.744 |
| *kdpFABC_BS_* | 18410 | 20983 | 0.590 |
| *kdpFABC_BS_-7* | 18412 | - | - |
| *kdpFABC_BS_-1* | 18412 | - | - |
| KdpE_DBD_ + *kdpFABC_BS_* | 30824 [1:1] / 42454 [2:1] | 47456 | 0.676 |
| KdpE_DBD_ +  *kdpFABC_BS_―7* | 30434 [1:1] / 42456 [2:1] | 32050 | 0.650 |
| KdpE_DBD_ +  *kdpFABC_BS_―1* | 30434 [1:1] / 42456 [2:1] | 34465 | 0.650 |

^a^ The
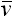
 used in calculations
